# Systematic offset of kV and MV localization systems as a function of gantry angle

**DOI:** 10.1120/jacmp.v12i1.3314

**Published:** 2010-11-09

**Authors:** John P. Mullins, Michael G. Herman

**Affiliations:** ^1^ Department of Radiation Oncology, Division of Medical Physics Mayo Clinic Rochester MN 55905 USA

**Keywords:** image‐guided radiotherapy, quality assurance, external beam radiotherapy, positioning accuracy, geometric uncertainty

## Abstract

Mechanical flex of the gantry and mounted imaging panels leads to systematic offsets in localization image isocenter as a function of gantry angle for linear accelerator‐mounted image guidance systems. Subsequently, object positions obtained from localization radiographs may be offset, resulting in greater target positioning uncertainty. While current QA procedures measure kV/MV image agreement, these measurements do not provide insight to apparent isocenter position for either single imaging system as a function of gantry rotation. This study measures offset as a function of gantry angle in kV and MV imaging systems on four treatment machines to investigate the magnitude of systematic offsets and their reproducibility between systems and machines, as well as over time. It is shown that each machine and energy has a reproducible pattern of offset as a function of gantry angle that is independent of kV/MV agreement, and it varies by machine. kV and MV offset ranges are on the order of 1.5 mm in the R/L and A/P directions, and 0.5 mm in the S/I direction. Variability of kV‐MV agreement is on the order of 0.7 mm. At certain angles, combinations of localization images could show a compounded offset of over 2 mm, exceeding the desired certainty threshold. Since these trends are persistent over time for each machine, online correction for image offsets as a function of gantry angle could improve the margin of positioning uncertainty.

PACS number: 87.55.Qr

## I. INTRODUCTION

The prevalence of target‐conforming beams, as well as the movement toward hypofractionation and dynamic arc IMRT, increases the need for accurate target positioning. Image guidance provides the necessary improvement in positioning accuracy.^(^ ^1^ ^)^ Setup images, whether kV radiographs or MV portal images, are typically acquired from multiple gantry angles, predominately cardinal orthogonals. Regardless of the imaging angles, it is known that gantry and imager rotation about the isocenter is not rigid and planar, which would be ideal.^(^ ^2^ ^)^ Depending on the magnitude of these variations, a target position determined from images from multiple angles may be offset significantly.

Several factors affect the hardware flex and image offset at various rotational positions. Head sag imposed by the weight of the gantry head, as well as similar sags in the supports for the MV image panel, kV image panel, and kV source all contribute to a net offset.^(^ ^3^ ^)^ For cone‐beam computed tomography (CBCT), correction of this offset through rotation is necessary for proper image reconstruction. Without rotational correction, CBCT images are blurred and contain increased streak artifacts.^(^ ^4^ ^)^ For this purpose, data is acquired at commissioning of a CBCT system to calibrate a software correction algorithm.^(^ ^5^ ^)^ Such corrections modify pixel‐lookup and back‐projection vectors to accommodate the effects of mechanical flex on reconstruction. Knowledge of the need for rotational CBCT correction predates commercially available kV IGRT systems; thus the principles of rotational flex correction generally apply to all similar systems and are not necessarily brand‐specific. This mechanical variation also exists for radiographs acquired with the kV and MV imaging systems but these may not be corrected as part of commissioning an IGRT system and may contribute to unwanted setup offsets.

Quality assurance (QA) procedures for kV imagers typically measure the registration between kV and MV image isocenters to ensure that agreement is within a stated threshold. The reference to isocenter is based on the MV portal imager QA, which registers a digital graticule to a physical graticule at one gantry angle. Thus, kV imager isocenter QA is only done relative to the MV image position and not to true mechanical isocenter. It is possible that a machinemay pass QA with a good agreement between kV and MV systems while both are offset from true room isocenter at various gantry positions. If this were the case, it would not be apparent through kV imager QA.

The purpose of this study is to examine the extent to which rotational offsets affect kV and MV radiographic images and to determine if these offsets are systematic and reproducible over time, across machines, or between the two systems. The significance to target localization is evaluated in the context of these uncertainties. A consistent systematic effect exhibited in the images could be software corrected as a function of gantry angle, leading to improved accuracy. This work also contributes to a better understanding of individual contributions to error in IGRT systems, which is useful for the successful clinical implementation of image‐guided positioning.

## II. MATERIALS AND METHODS

Gantry flex depends on numerous factors previously mentioned, and a compound effect can be measured by assuming rigid rotation of the image panels and tracking the apparent movement of a stationary target in images acquired at points of a rotation. Image isocenter was defined by a specific centroid pixel on the image panel for both kV and MV systems. The reported offset of the target on an image at a given angle compared to a reference image (at reference angle 180°) indicated the net effect of all mechanical flex on the apparent image isocenter for that angle.

### A. Data acquisition

To determine change in offset as a function of gantry angle, a radio‐opaque sphere (1 mm diameter) was placed at a fixed point near isocenter via lasers. Precise isocentric positioning was not required, as offsets were measured relative to a reference image for data acquisition of each system. KV and MV images were acquired in 20° steps over a full clockwise gantry rotation. To assess directional dependence, a counter‐clockwise rotation was performed for each clockwise rotation. This generated an equal number of clockwise and counter‐clockwise rotations for each setup. A data set corresponding to a single 360° rotation consisted of 19 measured 2D ordered pairs each (in the Cartesian space of the image) – one kV pair and MV pair corresponding to each 20° step. To investigate machine dependence, datasets were acquired for four different treatment machines (Clinac 21EX, Varian Medical Systems, Palo Alto, CA), each equipped with Varian On‐board Imager (OBI, version 1.6) and electronic portal imaging device (EPID) systems. For this paper, any comparison between “systems” or “energies” refers to a comparison between these two imaging systems, kV and MV respectively. Ten rotational datasets (5 CW, 5 CCW) were acquired for each machine over the course of six weeks to evaluate reproducibility over time.

### B. Geometric calculation

**Figure 1:**
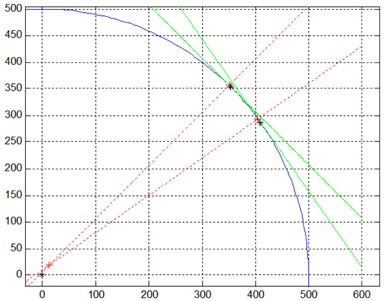
Geometric calculation process. Green lines represent imager panels on the circumference of gantry rotation (blue curve). The intersection of red dashed lines normal to imager panels indicates target position (red cross) relative to isocenter (black asterisk).

The 3D coordinate system used in this paper is the Right (R), Anterior (A) Superior (S) system with respect to the patient anatomy in head‐first supine orientation. Since 3D position information cannot be obtained from a single 2D radiograph, two consecutive images, 20° apart, were used to triangulate components of shift in the R/L and A/P directions. For this calculation, image panels were represented as tangent lines whose centers move about a circle of fixed radius (gantry rotation). On the acquired radiographic images, the on‐screen x‐axis position of the radio‐opaque sphere was back‐projected as a vector normal to the imager panels. Two vectors from images at 20° separation intersect at a point within the radius of gantry rotation that specifies the 2D position of the sphere on the R/L and A/P axes (Fig. 1).

The geometric operation was written as a MATLAB (The MathWorks, Natick, MA) function and was tested for user‐induced shifts of known magnitude and direction to verify its accuracy. The center angle between the two 20° images was the gantry position recorded with the resulting offsets. The S/I axis (the y‐axis measurement from acquired 2D ordered pairs) preserves its orientation independent of gantry angle throughout rotation, so an average S/I offset from the two 20° images was used in association with the center angle.

### C. Analysis

Data acquisition and geometric calculation resulted in 18 3D ordered pairs (L/R, A/P, S/I) in 20° gantry increments for the kV and MV imagers. The reference position of the sphere for each rotational dataset was the data acquired from the images at gantry position 180° (IEC 1217 scale). Offsets in target position measured at all other gantry angles were differences from the reference position for the given rotation. The datasets were tabulated and plotted to examine systematic trends in rotational offset for each machine and to compare offsets between different machines. Error bars were defined as one standard deviation. To compare kV/MV registration, the apparent positions of the target on kV and MV images for each gantry angle were compared and the differences plotted versus gantry angle for each machine. Error bar for the kv/MV comparison were determined by the sum of squares of one standard deviation from each energy.

## III. RESULTS

**Figure 2–7:**
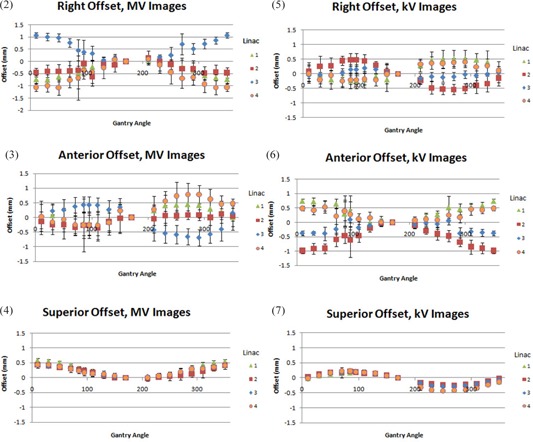
Averaged offsets for each energy, axis and linac. Numbers in the legend are linac designations. Each data point is an average of 10 rotational measurents (5 CW, 5 CCW). Error bars are one sigma.

Rotational data revealed a systematic trend in offset for both MV and kV imaging systems that was unique to each machine. CW and CCW rotations demonstrated no systematic differences for any machine. For the S/I offset, a systematic trend common to all four linacs was observed, although no common trend existed for the A/P and R/L axes. Figures 2–7 show average offsets in each direction for a full rotation. Numbers ‘1–4’ shown in the legends are designations of the four different linacs. These trends were reproducible over a period of six weeks.

The S/I offset is most consistent between linacs, with the smallest standard deviations on all machines and the smallest maximum magnitude of offset. Magnitude of offset in the S/I direction was approximately 0.5 mm for all linacs and both energies. There is a marked increase in standard deviation near the 90° and 270° gantry positions. This was most pronounced in the R/L direction for MV images and the A/P direction for kV images.

**Figure 8–11:**
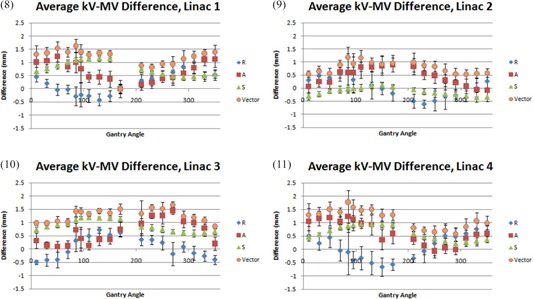
Difference in kV/MV apparent target positions. Error bars are the sum of squares of each modality sigma.

Difference in apparent target position between kV and MV images from each gantry position revealed systematic, reproducible patterns of isocenter agreement versus gantry angle. These patterns were specific to each machine and were different from the trends in single‐energy offset as a function of gantry angle. The four different machines had maximum vector kV‐MV differences of 1.63, 1.17, 1.66 and 1.77 mm occurring at gantry positions 85°, 85°, 270° and 85°, respectively. This indicates that kV‐MV vector difference is generally greatest near orthogonal angles. However, the gantry position‐dependent patterns of kV‐MV difference occur differently for each machine on each directional axis. For example, linac 1 vector difference at 85° is mainly constituted of approximately equal components in the A and S directions, with R difference being approximately 0. However, linac 2 vector difference at 85° is mainly comprised of approximately equal components in the R and A directions with S difference being approximately 0. These patterns are better visualized in Fig. 8–11.

## IV. DISCUSSION

Some standards for QA evaluation of kV image‐based localization systems measure the agreement of the kV isocenter with the MV isocenter as represented by the digital graticules.^(^ ^6^ ^)^ While the results of all measurements in this work suggest the total variation resides within about a 2 mm window, it is clear from comparing the kV‐MV agreement in Figs. 8–11 to the rotational offsets of each energy shown in Figs. 2–7 that the trends are systematic and vary significantly by energy, machine and axis. Further, the rotational variation is not assessed by current kV imager QA procedures. For example, linac 2 passes the monthly QA threshold with a kV‐MV difference of 0.54 mm at 0°. At any other angle, the kV‐MV difference changes, as well as the offset in image position for each system, and the observed offset would be the sum of kV offset, MV offset and kV‐MV difference. If conditions were such that offsets reached one standard deviation based on plots 3, 6 and 9, a lateral MV and A/P kV image from 90° gantry position would yield a disagreement of 2.52 mm in the anterior direction alone.

Image offsets due to rotational flex may be small contributions to overall positioning uncertainty, but their values are quantifiable and repeatable and may not be insignificant in all cases. Although maximum offset in any direction is only approximately 1 mm, the common use of orthogonal images for positioning make awareness of the offset and higher standard deviation at orthogonal angles important. These data suggest that such systematic offsets for localization radiographs are unique to each treatment machine and are consistent over time. While it is routine to measure and correct the offsets for more accurate CBCT reconstruction, it would also be possible to implement a correction algorithm for stationary radiographs, improving geometric accuracy of kV localization. Commissioning and QA of OBI and EPID systems would then involve a step‐and‐shoot measurement sequence that could be logged and calibrated via a correction algorithm for isocenter position as a function of gantry angle and then monitored.

Offsets in this study were shown to persist on each machine and axis for the period of the study, suggesting stability (six weeks). While the magnitude of the offsets is on the order of 1 mm, under some circumstances contributions to the localization error could exceed 2 mm. The relative ease of making a correction along with the stability of the offsets suggests correction should be carried out to reduce uncertainty in image guidance. After initial calibration, follow‐up measurements should be made as part of periodic (quarterly, annual) quality assurance, so that the correction calibration obtained at commissioning can be reviewed and adjusted. Measurements should also be repeated after servicing the kV tube or detector panels.

Also of note is the increased standard deviation at orthogonal gantry head positions (90° and 270°). The prevalence of orthogonal images for positioning makes the understanding of positioning accuracy at these specific angles more important, especially if no systematic correction algorithm is in place. It may be valid to investigate potential improvements in positioning and treatment achievable by using images taken from angles of planned treatment fields instead of the standard setup orthogonals. Similarly, dynamic arc therapies may be more accurate if setup images are taken from milestone positions of the arc, such as start, stop or center angle positions.

Whether or not rotational offset is used for systematic correction or the planning of setup image angles, the information available from such datasets can be useful to the treatment team for a comprehensive understanding of image‐guided radiotherapy capabilities and limitations. The measurement of this offset for each kV/MV imager‐equipped linac can be assessed to ensure all offsets fall within a particular acceptance threshold. Magnitude of rotational image uncertainty could also be factored into departmental PTV margin guidelines.^(^ ^7^ ^,^ ^8^ ^)^

## V. CONCLUSIONS

Users of image‐guided localization systems should be aware of the systematic offsets inherent in kV and MV imaging systems as a function of gantry angle. Rotational offsets of either imaging system are not assessed by current standard QA procedures and cannot be derived from measurements of kV/MV agreement. Despite the seemingly small magnitude of systematic rotational offsets, these offsets do exist, are reproducible over time and are unique to a given treatment machine. Once measured, a correction could be applied to address these offsets in the same manner already ubiquitous for CBCT. The quantification and reduction of uncertainty in the radiotherapy process contributes to improvement in accuracy, and can potentially improve the quality of treatment.
